# Feasibility and safety of expandable mediastinoscopic and laparoscopic radical esophagectomy

**DOI:** 10.3389/fonc.2023.1110962

**Published:** 2023-04-19

**Authors:** Weidong Zhang, Dong Cui, Kefeng Shi, Maolin Chen, Binbin Zhang, Rulin Qian

**Affiliations:** ^1^ Department of Thoracic Surgery, Henan Provincial Chest Hospital, Zhengzhou University, Zhengzhou, Henan, China; ^2^ Henan Provincial Mediastinoscope Diagnosis and Treatment Center, Zhengzhou, Henan, China

**Keywords:** expandable mediastinoscope, radical esophagectomy, esophageal cancer, feasibility, safety

## Abstract

**Background:**

At present, minimally invasive radical esophagectomy is the main surgical method for esophageal cancer treatment, but it has inherent limitations. We have developed a novel method of radical esophagectomy without thoracotomy to improve this situation, namely, by using EMLE. We evaluated the feasibility and safety of expandable mediastinoscopic and laparoscopic radical esophagectomy (EMLE) through a retrospective analysis.

**Methods:**

From January 2019 to June 2022, we successfully performed 106 cases of radical resection of esophageal cancer with this new surgical technique, gradually improved the surgical path, and recorded the perioperative data and postoperative complications of all patients.

**Results:**

The operation was successfully performed in all patients except for two patients who required a switch to open surgery. The mean operation time was 171.11 ± 33.29 min and the mean intraoperative blood loss was 93.53 ± 56.32 ml. The mean number of removed lymph nodes was 23.59 ± 5.42. The postoperative complications included pneumonia (3.77%), recurrent laryngeal nerve palsy (1.89%), anastomotic leak (14.15%), pleural effusion (5.66%), chylothorax (2.83%), and reoperation (4.72%). All complications were graded I–III per the Clavien–Dindo classification. No perioperative death was recorded.

**Conclusion:**

Expandable mediastinoscopic and laparoscopic radical esophagectomy is feasible for radical resection of esophageal cancer, with good therapeutic effect and safety. Because of its minimal impact on patients and convenient operation, it is a novel surgical option for patients with esophageal cancer and is expected to become a standard surgical method for radical esophagectomy in the future.

## Introduction

Esophageal cancer is a common tumor of the digestive system. Its morbidity and mortality rank seventh and sixth in the world, respectively. At present, radical resection remains the first and main treatment choice for esophageal cancer (1). The surgical methods of esophageal cancer include traditional thoracotomy and endoscopic minimally invasive surgery. Traditional radical esophagectomy requires thoracotomy into the thoracic cavity and laparotomy into the abdominal cavity, which greatly destroys the thoracic and abdominal structures. It causes significant trauma to patients and is associated with many postoperative complications, with an incidence of 20–50% (2), thereby seriously affecting patient prognosis. Video-assisted thoracoscopic and laparoscopic radical esophagectomy (VATLE) has been rapidly promoted, because it can greatly reduce the surgical trauma of patients and promote rapid recovery (3). At present, it is the first minimally invasive surgical method of choice for esophageal cancer and is recommended by the NCCN guidelines (4). However, this operation method also has some limitations, such as the tedious procedure of transthoracic surgery, the need to change body position during the operation, the long operation time, the considerable interference to the heart and lung of the patients, and the need for patients to have a good cardiopulmonary function reserve. In addition, if there are adhesions in the chest, previous history of lung disease, chest deformities, or other factors affecting the transthoracic approach, the procedure of VATLE will be very difficult. In patients whose cardiopulmonary function cannot tolerate the thoracoscopic surgery, the surgical approach will have to be abandoned.

Because of these problems, some surgeons began to try to change the original surgical methods to diversify the surgical methods of esophageal cancer (5–9), so that thoracic surgeons can have more choices for patients with esophageal cancer under different conditions and improve the overall prognosis. Based on the development of mediastinoscopic technology, minimally invasive esophageal surgery, and video-assisted mediastinoscope, our team successfully developed a new surgical method in 2019, namely the expandable mediastinoscopic and laparoscopic radical esophagectomy (EMLE) approach. We have completed 106 cases of radical resection of esophageal carcinoma using this novel surgical technique from January 2019 to June 2022. In this report, we analyze the therapeutic efficacy and safety of this surgical method in these patients.

## Materials and methods

### Patients

A total of 106 patients with esophageal cancer undergoing EMLE for radical esophagectomy from January 2019 to June 2022 in our hospital were included. The inclusion criteria for this type of surgery were: (1) gastroscopic and pathological diagnosis of esophageal squamous cell carcinoma, clinical stage I–IIIA; (2) age: 18–80 years; (3) cardiopulmonary function could tolerate surgical treatment; (4) patients did not have other cancers; and (5) patients voluntarily participated in the clinical trials. The exclusion criteria were: (1) cervical esophageal squamous cell carcinoma, and (2) patients refused surgical treatment. The study was approved by the Ethics Committee of Henan Provincial Chest Hospital (2019-03-008), and written informed consent was obtained from all patients for the publication of any potentially identifiable images or data included in this article.

**Figure 1:**
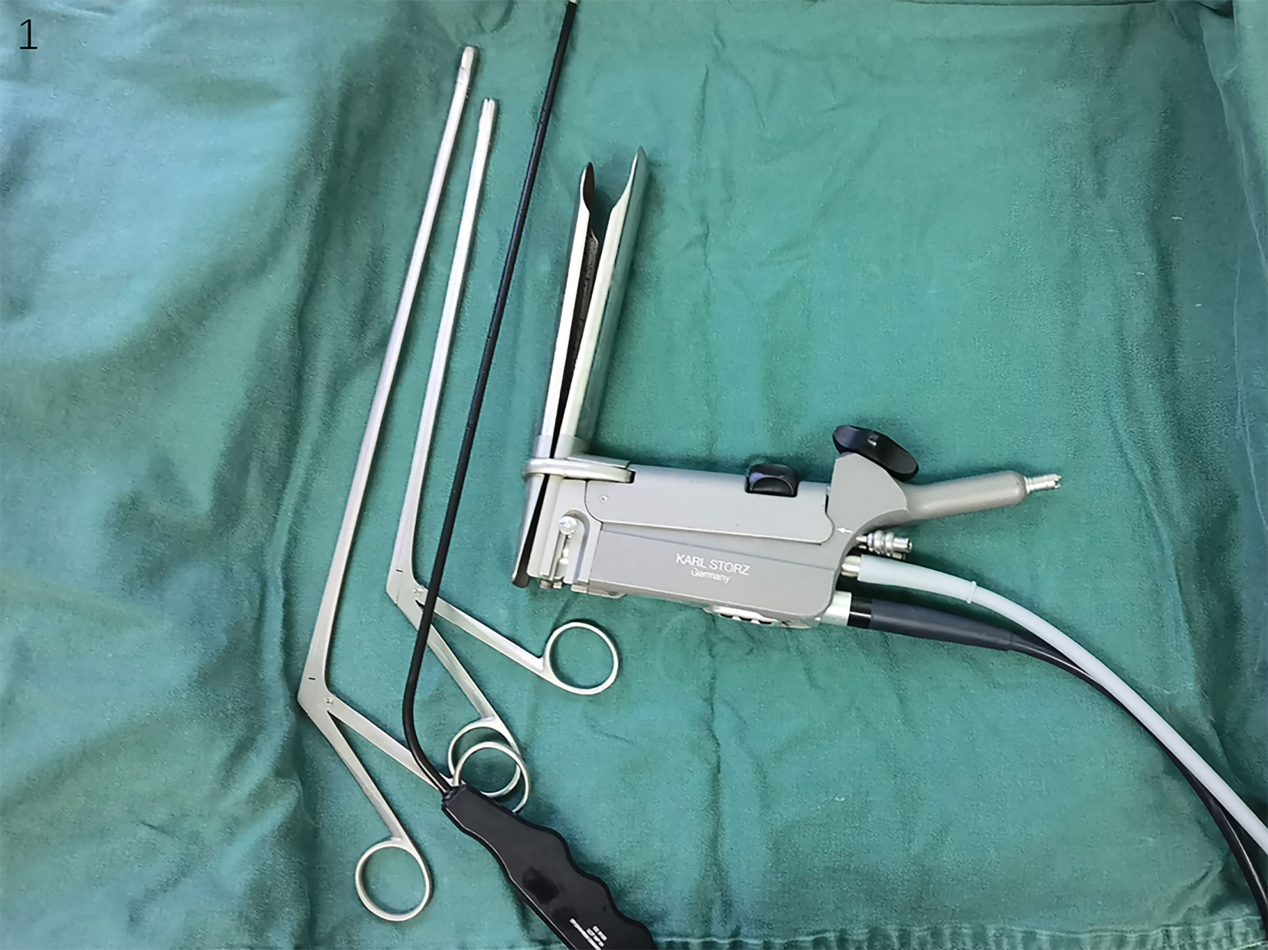
The instruments of expandable mediastinoscope.

All patients underwent EMLE with the new expandable television mediastinoscope system (10972SP, STORZ, KARL STORZ SE & Co. KG, Germany) (**Figure 1**), and the concept of “four channel lymph lymphadenectomy” was used to explore and dissect the lymph nodes.

### Surgical procedure

**Figure 2:**
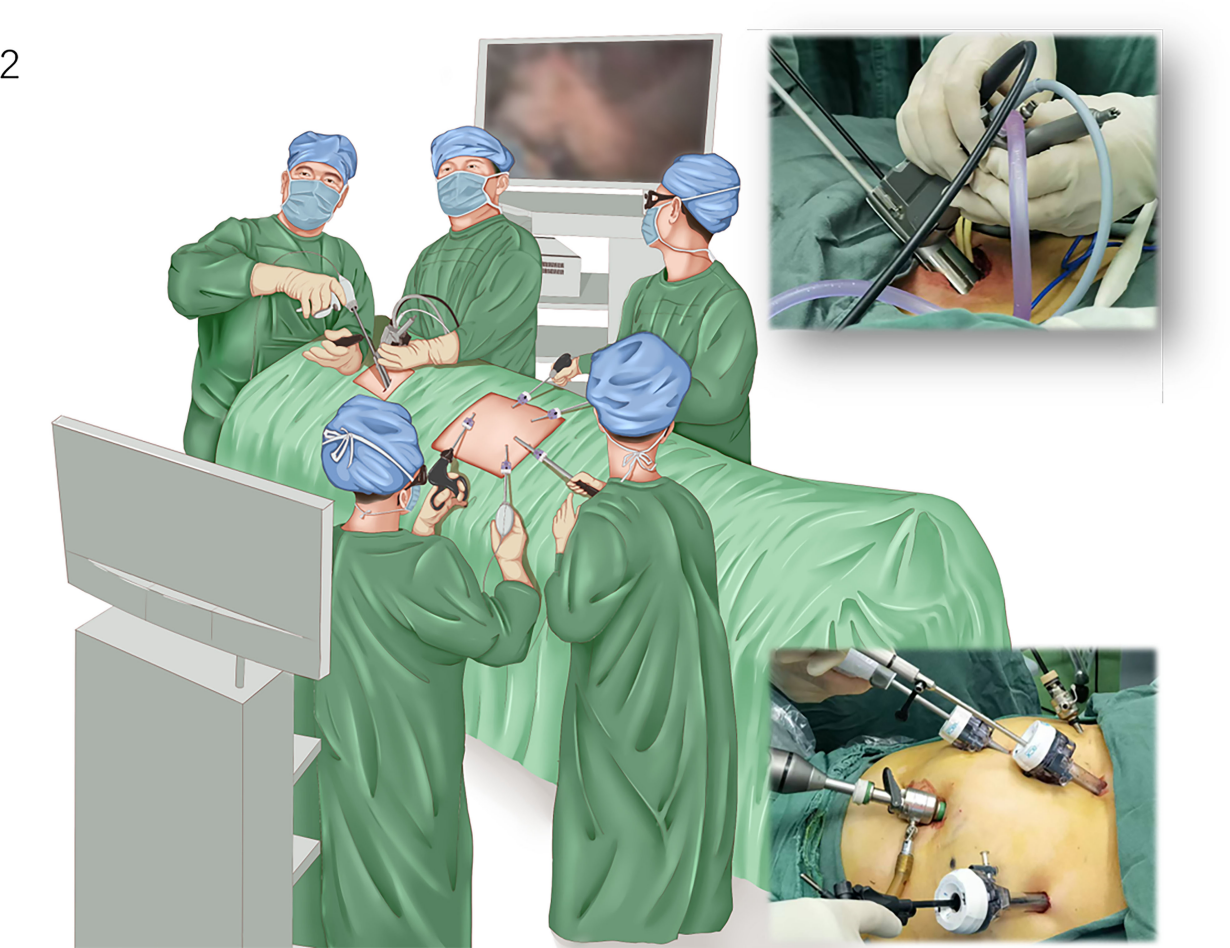
Operative setting for video-assisted mediastinoscopic and laparoscopic radical esophagectomy. The patient is placed in the supine position with the neck properly elevated. The mediastinoscope group comprised two doctors located next to the patient’s head, and the laparoscopic group comprised three physicians on both sides of the patient.

The EMLE procedure was initiated with single-lumen intubation under general anesthesia. The patient was placed in the supine position, the neck properly elevated, and both lungs ventilated. During the operation, there were two groups of doctors—the mediastinoscopy group and the laparoscopy group. The former group comprised two doctors, and the latter group comprised three physicians on both sides of the patient. The operating room setup is shown in **Figure 2**.

#### Step 1: cervical mobilization of the esophagus and lymphadenectomy

**Figure 3:**
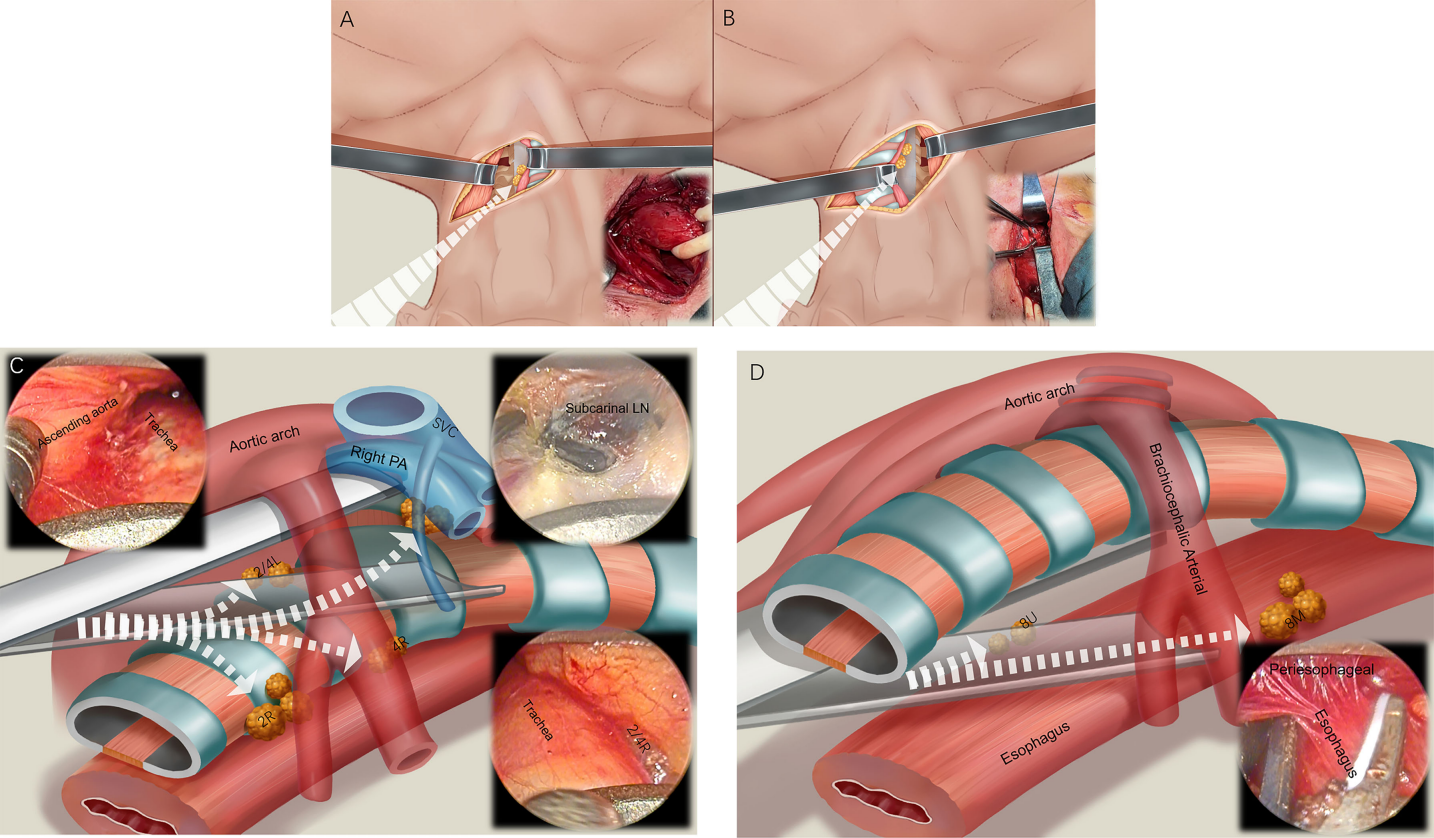
Four channel lymphadenectomy. After the 5-cm arc neck incision is made, the first channel is entered to dissect the left paraesophageal lymph nodes and 1L lymph nodes **(A)**. Then, the second channel is used to dissect the right paraesophageal lymph nodes and 1R lymph nodes **(B)**. The pretracheal space (third channel) is then entered, and the 2R, 4R, 7, 2L, and 4L lymph nodes are dissected **(C)**. Finally, we enter around the esophagus (fourth channel), dissect the 8U and 8M lymph nodes, and free the esophagus **(D)**.

A 5-cm arc incision was made from the front edge of the left sternocleidomastoid muscle to the anterior midline of the neck, and the space between the muscle and the left side of the tracheoesophagus was separated into the first channel (i.e., the space on the left side of the neck and the esophagus). The left recurrent laryngeal nerve (RLN) was identified and protected, and the left paraesophageal cervical lymph nodes and left supraclavicular lymph nodes (left paraesophageal lymph node and 1L) were dissected. An esophageal band was placed at the cervical esophagus, which was pulled to the left, and the second channel (i.e., the space on the right side of the cervical esophagus) was entered into at the right edge of the tracheoesophagus. The right paraesophageal cervical lymph nodes and the right supraclavicular lymph nodes were dissected (right paraesophageal lymph node and 1R) (**Figures 3A, B**).

#### Step 2: mediastinoscopic mobilization of the upper and middle thoracic esophagus and lymphadenectomy

The expandable mediastinoscope was placed in front of the trachea, entering the third channel (i.e., the pretracheal space), and reaching the right edge of the trachea. The lymph nodes of the right RLN (2R, 4R) were dissected (**Figure 3C**). Moving forward to the subcarinal space of the trachea, the subcarinal lymph nodes (7) and, reaching the left edge of the trachea, the left RLN lymph nodes (2L, 4L), were dissected. The mediastinoscope was placed around the esophagus, and the fourth channel was entered into (i.e., the paraesophageal space). We used the mediastinoscope to expand and slowly advance forward, freeing around the thoracic esophagus to the level below the carina, to meet the laparoscopy procedure. During this process, the paraesophageal lymph nodes (8U, 8M) were cleaned (**Figure 3D**).

During the operation, the assistant used the esophageal strap to pull the esophagus in different directions according to the needs of the operator, and used the mediastinoscope to expand and slowly advance forward. The thoracic surgeon operated using two instruments—a suction device and an ultrasonic scalpel.

#### Step 3: laparoscopic mobilization and formation of a gastric conduit and lymphadenectomy

The abdominal stage operations were performed under a 12 mmHg CO2 pneumoperitoneum, including formatting a gastric conduit, dissociating the middle and lower thoracic esophagus through the esophageal hiatus, and dissecting the 8L, 9, and 15–20 lymph nodes, as described in previous studies (10, 11). The cervical mediastinoscopy group and abdominal laparoscopy group performed their respective operations simultaneously.

#### Step 4: gastroesophageal anastomosis

The fabricated tubular stomach was pulled up to the cervical incision via the mediastinal cavity, and an end-to-side gastroesophageal anastomosis was performed using a circular stapler. The surgery was concluded after the placement of a neck drainage strip around the anastomotic site, a mediastinal drainage tube through the abdominal incision, and stomach tube and feeding tube was placed in the stomach and jejunum respectively, followed by closure of the neck and abdominal wounds.

### Postoperative management

All patients were given parenteral nutrition support after the operation, checked the neck incision and paid attention to body temperature changes every day. If there were no abnormality, started the liquid diet around 7-10 days, and removed the feeding tube. The stomach tube was removed after the gastrointestinal motility became normal (usually about 3 days). The mediastinal drainage tube was usually removed when the drainage volume was less than 50ml, and the color was free of abnormal features such as blood and chyle. After resumption a normal liquid diet, the discharge criteria were met. Reviewed and arranged follow-up treatment, including adjuvant chemotherapy, one month after discharge.

### Statistical analysis

Continuous data were presented as the mean± standard deviation (SD). Categorical data were expressed as number (percentage). All statistical analyses were performed using GraphPad Prism version 8.30 (GraphPad Prism, GraphPad Software, San Diego, USA).

## Results

A total of 106 patients (71 male and 35 female) with esophageal cancer were enrolled in the study. The mean age was 65.39 ± 8.31 years (median [IQR]: 66 [59, 71] years). Among them, 55 patients (51.89%) had other diseases including hypertension; heart disease (coronary heart disease, congenital heart disease, valvular heart disease, arrhythmia); diabetes mellitus; chronic lung disease (pulmonary tuberculosis, pleurisy, bronchiectasis, emphysema, chronic tracheitis, interstitial pulmonary fibrosis); cerebrovascular disorder; and liver disease. The patients’ height range was 90–178 cm (median [IQR]: 165 [160, 170] cm), and mean height was 164.39 ± 9.56 cm. All patients were examined by a gastroscope before the operation to determine the location and pathology of the tumor, which was mainly located in the middle of the chest. The pathological type of the tumor was squamous cell carcinoma. The general characteristics of all patients are summarized in **Table 1**.

**Table 1:** Patient Demographic and clinical characteristics.

	n=106	%
Sex		
Male/Female	71/35	66.98/33.02
**Age**(Year) (Mean ± SD)	65.39 ± 8.31	
**Comorbidity** (No/Yes)	51/55	48.11/51.89
HT/HD/DM/CLD/CD/LD	32/23/13/11/7/3	
Tumor location		
Upper/Middle/Lower	17/59/30	16.04/55.66/28.30
**Height**(cm) (Mean ± SD)	164.39 ± 9.56	
Clinical T		
T1/T2/T3/T4	32/28/39/7	
Clinical N		
N0/N1/N2	73/27/6	
Clinical M		
M0/M1	105/1	
Clinical stage		
I/II/III/IV	34/36/35/1	32.08/33.96/33.02/0.94
**Preoperative neoadjuvant chemotherapy**(No/Yes)	81/25	76.42/23.58

HT, hypertension; HD, heart disease(coronary heart disease, congenital heart disease, valvular heart disease, arrhythmia); DM, diabetes mellitus; CLD, chronic lung disease (pulmonary tuberculosis, pleurisy, bronchiectasis, emphysema, chronic tracheitis, interstitial pulmonary fibrosis); CD, cerebrovascular disorder; LD, liver disease.

Among all patients, 104 underwent complete EMLE. One patient required conversion to thoracotomy because of an injury to the left main bronchial membrane, and one patient required conversion to laparotomy because of uncontrollable abdominal bleeding. Among all the successful operations, four patients underwent other operations at the same time, including two cases of coronary artery bypass graft surgery and one case each of thyroidectomy and appendectomy. The mean operation duration was 176.71 ± 52.26 min, and the mean intraoperative blood loss was 101.35 ± 79.40 ml. A total of 23.62 ± 6.22 lymph nodes were dissected during the operation; one patient had cervical lymph node metastasis. Patients were classified into stages I (32.08%), II (33.96%), III (33.02%), and IV (0.94%). The above information is presented in further detail in **Table 1**. **Table 2** provides details of postoperative complications. A total of 30 patients (28.30%) had postoperative complications, including pneumonia (3.77%, n=4); RLN palsy (1.89%, n=2); anastomotic leak (14.15%, n=15); pleural effusion (5.66%, n=6); and chylothorax (2.83%, n=3). Five patients (4.72%) required reoperation, among whom two underwent mechanical hemostasis for mediastinal bleeding and abdominal bleeding, one underwent thoracic duct ligation because of severe chylothorax, and the remaining two required debridement and re-repair because of severe cervical anastomotic leak. There were no perioperative deaths in this study. All patients only retained a mediastinal drainage tube to drain the effusion from the mediastinal and abdominal cavities after surgery. The mean indwelling time of mediastinal drainage tube was 5.70 ± 3.81 days. After the gastrointestinal nutrition tube was removed, the patient could be discharged if they ate normally. The mean postoperative hospital stay was 25.33 ± 17.88 days.

**Table 2:** Perioperative outcomes.

	n=106	%
**Operation time** (min) (Mean ± SD)	176.71 ± 52.26	
**Blood loss** (ml) (Mean ± SD)	101.35 ± 79.40	
**Simultaneous surgical treatment** (n)**^a^**	4	3.78
**Conversion to open surgery** (n)**^b^**	2	1.89
**Removal time of MDT**(d) (Mean ± SD)	5.70 ± 3.81	
**Postoperative hospital stay** (d) (Median)	19.5	
**No. of the resected LN** (Mean ± SD)	23.62 ± 6.22	
**Postoperative complications** (n)	30	28.30
Pneumonia	4	3.77
RLN palsy	2	1.89
Anastomotic leak	15	14.15
Pleural effusion	6	5.66
Chylothorax	3	2.83
Reoperation	5	4.72
**In-hospital mortality** (n)	0	0.00
**R0 resection** (n)	106	100.00

a: Four patients underwent other simultaneous operations, including two cases of coronary artery bypass graft surgery and one case each of thyroidectomy and appendectomy.

b: Two patients were converted to open surgery, and their operation time and blood loss were not included in the statistical analysis due to large deviation. One patient required conversion to thoracotomy because of an injury to the left main bronchial membrane, and one patient required conversion to laparotomy because of uncontrollable abdominal bleeding.

MDT, mediastinal drainage tube; LN, lymph nodes; RLN, recurrent laryngeal nerve.

## Discussion

The surgical methods for esophageal cancer include thoracotomy and endoscopic minimally invasive surgery. VATLE is the mainstream minimally invasive surgery for esophageal cancer at present. However, because this operation requires thoracic surgery, it is limited in clinical application, resulting in the loss of surgical opportunities for some patients. Radical resection of esophageal cancer via the mediastinal cavity has become a new therapeutic choice. This operation can free the esophagus and remove lymph nodes in the mediastinal cavity, which is a complete radical resection of esophageal cancer. Transmediastinal radical resection of esophageal cancer has also become a new option, which can be used for esophageal dissociation and mediastinal lymph node dissection and is a complete radical resection of esophageal cancer.

At present, the methods of transmediastinal esophagectomy include inflatable mediastinoscopic and laparoscopic radical esophagectomy (IMLE), and EMLE. IMLE was a new surgical method developed and improved by Mori (5), Fujiwara (6), Daiko (7), and Wang (10), whose surgical safety and efficacy have been preliminarily verified. However, in essence, IMLE was the application of ordinary endoscopic instruments in the mediastinal cavity, not the application of a mediastinoscope in the true sense, and hence has the following limitations: the neck requires double incisions on the left and right; the operation establishing a closed single-hole incision in the neck is complicated; simultaneous inflation of the mediastinum and abdominal cavity increases the occurrence of adverse events such as instability of the respiratory and circulatory system and subcutaneous emphysema; and, given that the mediastinal cavity needs to be inflated, it is difficult to expose the surgical area in case of unexpected circumstances such as bleeding, which would increase the risk of surgery.

With the development and improvement of mediastinoscopic instruments and mediastinoscopic techniques, we explored the surgical methods for esophageal cancer and successfully carried out a new surgical method called EMLE. This approach made full use of the characteristics of the expandable mediastinoscope to expand the space between the esophagus and the surrounding tissues in the mediastinum, increase the operation space, and increase the tissue traction to assist the operation. In this case, only two surgical instruments (a suction device and an ultrasonic scalpel) were generally used to complete most operations, which significantly improved the operation space, increased visibility of the surgical field, reduced the surgical risk, and increased the overall safety of the procedure. These advantages addressed the limitations of radical resection of esophageal cancer under an inflatable mediastinoscope.

According to the results of blood loss, operation time, and complications in our operation group, EMLE was both safe and reliable. Because the operation process did not require the patient to turn over, and the operative field was clearly displayed under the expandable video mediastinoscope, EMLE was superior to the perioperative results of thoracotomy and VATLE in terms of surgical blood loss and operation time (12). It was also superior to thoracotomy and VATLE, which have been reported in the past; this was helped by the fact that transmediastinal endoluminal surgery can significantly reduce the impact on the lungs (2, 13). We summarized perioperative data such as postoperative complications, conversion rate to thoracotomy, in-hospital mortality, and postoperative hospital stay, and did not find that EMLE was inferior to other surgical methods, even robot-assisted surgery (8, 14). In terms of the incidence of RLN palsy and chylothorax after surgery, our data were better than some of the reported literature data (12), which may be because of two reasons: (i) the clear vision and wide exposure of the expandable mediastinoscopic system during the surgery, and (ii) the rich experience accumulated over a prolonged period by our medical center with respect to operating using a mediastinoscope. In our study group, two patients underwent coronary artery bypass grafting combined with EMLE, which has been proved to be both safe and feasible in the treatment of esophageal cancer patients with coronary heart disease.

In the past, the completeness of lymph node dissection has always been a key part of the questionable transesophageal resection via the mediastinum. However, thanks to the clear visual exposure under the expandable mediastinoscope and the application of the “four channel lymphadenectomy” that we first developed, we could complete the exploration and dissection of the three-field lymph nodes. In fact, because the lymph nodes around the left RLN can be detected in the mediastinal cavity under the expandable mediastinoscope, the clearance rate was better than that of the unilateral thoracic surgery, and our technique was not inferior to other surgical methods regarding the total number of lymph nodes, and was unlikely to increase the probability of RLN palsy (15).

Compared with VATLE, EMLE was only performed in a relatively narrow mediastinal cavity, which may be difficult to dissociate from the tumor invading the outer membrane of the esophagus or tissues outside the esophagus, and increasing the risk of surgery. Therefore, we do not recommend this operation for patient with clinical stage T4, but these patients can be re-evaluated after preoperative neoadjuvant chemotherapy. However, there was no strict limit on the number of lymph node metastasis. Most of the resectable lymph nodes near the esophagus or in the mediastinum can generally be completely removed. Hence, the recommended staging of esophageal cancer in EMLE was T1-3N0-2M0. Of course, skilled and experienced doctors can appropriately expand the scope of application of EMLE. Given the feature of non-transthoracic operation, EMLE can reduce the dependence on patients’ chest condition and cardiopulmonary functions, and increase the surgical opportunity of some patients with poor cardiopulmonary function. Because the maximum length of the expandable mediastinoscope was 15 cm, the height of the patient may also be a key factor in determining whether EMLE was feasible. At the beginning of EMLE, we tried to select subjects shorter than 175 cm. Later, we used 3D-laparoscope to free the lower thoracic esophagus through the esophageal hiatus, thereby increasing the height of the free esophagus. We found that it was still safe and reliable when patients’ height increased to 178 cm. Ultimately, we suggested that for inexperienced surgeons, the lower the patient’s height, the higher the success rate.

We acknowledge that this study has some limitations. As EMLE is a brand-new surgical method, it has thus far only been carried out in our clinical center on a large scale. Although we have data to verify the safety and therapeutic effect of EMLE (11), we will still need more reliable verification of mid- and long-term efficacy. We have carried out a currently conducting comparative studies with other common surgical methods such as thoracotomy and VATLE to obtain more reliable evidence. We are also planning to conduct prospective randomized controlled trials to more comprehensively evaluate the safety and mid- and long-term outcomes of EMLE.

In conclusion, our research results indicate that EMLE is a reliable new surgical method and a feasible non-transthoracic treatment option for esophageal cancer. Perioperative safety and lymph node dissection could achieve or even be superior to other surgical methods, and the dependence on cardiopulmonary conditions in patients with esophageal cancer was lower. We believe that as long as we can fully understand the anatomical structure of the mediastinum and continue to learn more relevant mediastinoscopy techniques, we can successfully carry out EMLE, so that patients with esophageal cancer have more choices than at present with regard to surgical methods.

## Data availability statement

The original contributions presented in the study are included in the article/**Supplementary material**. Further inquiries can be directed to the corresponding author.

## Ethics statement

The studies involving human participants were reviewed and approved by The Ethics Committee of Henan Provincial Chest Hospital. The patients/participants provided their written informed consent to participate in this study. Written informed consent was obtained from the individual(s) for the publication of any potentially identifiable images or data included in this article.

## Author contributions

WZ and RQ designed the entire study. WZ, DC, KS, BZ and MC conducted patient clinical management and sample collection. WZ, MC and DC analyzed the data. WZ wrote the manuscript. All the authors read and approved the final version of the manuscript for submission.

## Supplementary material

The The Supplementary Material for this article can be found online at: https://www.frontiersin.org/articles/10.3389/fonc.2023.1110962/full#supplementary-material
